# Algorithm based on normal coordinate vectors with 16 segments for the data fusion from hand-written Arabic text implemented with MATLAB

**DOI:** 10.7717/peerj-cs.705

**Published:** 2021-09-09

**Authors:** Said S. Saloum, Iván García-Magariño

**Affiliations:** 1 College of Computer and Information Sciences, Jouf University, Sakaka, Saudi Arabia; 2 Department of Software Engineering and Artificial Intelligence, Complutense University of Madrid, Madrid, Spain; 3 Instituto de Tecnología del Conocimiento, UCM, Madrid, Spain

**Keywords:** Handwriting recognition, Normal coordinate vectors, MATLAB, Data fusion

## Abstract

Hand-written text recognition is useful for interpreting records in different fields such as healthcare, surgery and police in which professionals may avoid technical equipment and prefer writing notes on paper. In order to perform data fusion from different data sources, handwriting automatic recognition involves barriers such as different ways of writing letters and deformation due to many reasons. This work presents a novel handwriting recognition approach based on the application of coordinate vectors to find similarities in different kinds of deformations. In particular, it has been implemented using 16 segments in order to distinguish all the particularities in matching the new text considering a dataset with a machine-learning approach. The implementation of this approach with MATLAB shows promising results with accuracy of 92.8% for with ensemble and bagged trees, after analyzing 22 possible combinations of machine learning and processing techniques.

## Introduction

Data fusion requires many different steps for extracting similar data from different sources. This sources involves matching similar features from different sources. For instance, dimensional reduction can be applied for extracting similar features (Zhang et al., 2021). Some works focus on the algorithms for data fusion while others focus on the data structure (Azcarate et al., 2021). Regarding the techniques applied, most methodologies use ML covering from using deep neural networks (Zhang et al., 2021) for covering stream of data from IoT or different ML methods for analyzing images like in information fusion of Earth observation (Salcedo-Sanz et al., 2020). However, to the best of authors’ knowledge the literature lacks proposing methodologies for the information fusion from Arabic hand-writing.

Hand-written recognition has allowed interpreting and automatically processing hand-written documents facilitating the digital interaction with previous documents. It also allows certain professionals to make notes without needing a computerized device, for its latter processing. Hand-written text recognition has been addressed in many different ways. For example, hand-written character recognition has been implemented with fuzzy and artificial neural-network techniques (Tay & Khalid, 1997). Genetic programming has been used for identifying hand-written digits (Parkins & Nandi, 2004). Soft computing techniques have been used for applying feed-forward neural network for recognition of hand-written English alphabets. Thus, ML has been widely used combining it with different techniques for contributing to the processing either avoiding non-relevant aspects or narrowing the search space for facilitating processing similar hand-written patterns.

Coordinate vectors have supported many different fields by representing space positions as a linear combinations of vectors. For instance, coordinate vectors have been used in chemistry for explaining and predicting reactions, such as polyhedral container molecules (Browne et al., 2013). Coordinate vectors have also been useful in theoretical biology for binding shaping the space binding antibodies and antigens (Lapedes & Farber, 2001). Furthermore, coordinate vectors have been used for medical image reconstruction (Hou, 2020), using this representation for improving the application of ML. More concretely in the recognition field, coordinate vectors were used for iris recognition in image analyses (Daugman, 2007). These works reveal the benefits of not restricting to orthogonal representation of space in order to match different realities in nature. However, to the best of our knowledge, hand-written recognition systems have not benefited from the application of coordinate vectors for application of ML.

One of the main challenges of analyzing Arabic handwriting is the pre-processing as stated in the literature (Jayech, Mahjoub & Amara, 2016). We discarded the common Optical Character Recognition (OCR) techniques due to their known low accuracy rates when applied to handwriting (Pramanik & Bag, 2018).

The current work proposes a novel approach that integrates the use of coordinate vectors in the application of ML for hand-written recognition for identifying similar writing patterns in different spaces with coordinate vectors.

The remainder of the paper is organized as follows. The next section introduces the most relevant related work highlighting the gaps covered in this work. ‘Method for handwriting recognition’ describes the method for performing hand-written recognition using the novelty of coordinate vectors in this field. ‘Results’ shows the experimentation using this novel approach. ‘Discussion’ discusses the most relevant aspects. Finally, ‘Conclusion and future work’ mentions the conclusions of this work and some future research lines.

## Related work

In the context about analysis of handwriting, we have classified the existing related works into (a) data fusion applications, (b) artificial intelligence works, and (c) recognition of Arabic.

In the group of data fusion applications, there is a ranking of data fusion methods applied to online handwriting information retrieval (Saldarriaga, Morin & Viard-Gaudin, 2010). This ranking is based on the fact that information retrieval can be improved by applying data fusion techniques in handwriting documents. Data fusion is relevant specially for extracting information from noisy texts. In this field, Arkenbout, De Winter & Breedveld (2015) presented a robust hand motion tracking system based on data fusion from a data glove and a Kinect camera. This data fusion of different devices supported the detection of hand motion with a high accuracy. Moreover, the multi-modal data fusion has been used for authenticating users through their handwriting (Ji et al., 2021). They extracted features from different devices and merged this information for verifying that a document was written by a certain person avoiding forgery attacks. In this context, the current work also uses data fusion in the context of handwriting, but its main novelty is that it focuses specifically in Arabic writing and its different particularities.

In the group of artificial intelligence works, hand-written recognition has been addressed in many different ways in the literature over the time. Most of the recognition systems use ML for associating characters or group of characters with existing ones from a corpus. ML covers a great variety of methods such as artificial neural networks, k-nearest neighbors (KNN) and support-vector machines (SVM). One of the main differences among the existing works on this field is the way of preprocessing or representation images for efficient

Some works have focused on the identification of features and combining them for hand-writing recognition. For instance, Hassan et al. (2014) presented an identification system of multiple feature based on multi-kernel learning. They used a genetic algorithm and proved the efficacy of their framework for using combination of features. The combination of features has been applied across different languages.

Regarding the application of ML techniques, one of the most advanced and recent hand-written recognition systems uses a deep learning model (Khan et al., 2021). In particular, they used a convolutional neural network using squeeze and excitation blocks. Other works like the one from Yang et al. (2020) show that dimensionality reduction is key for proper hand-writing recognition. More concretely, they showed how small sample entropy was useful for training writing recognition systems discarding irrelevant dimensions. The dimensionality reduction has been widely found useful in ML areas in which there is a large variety of information, as also proven in the context of real-estate market with living units with many features (García-Magariño, Medrano & Delgado, 2020). The current work aligns with these works since coordinate vectors help forming the most relevant dimensions.

In the field of handwriting recognition, there is an up-growing interest in air handwriting. Unsupervised domain adaptation has been applied for recognizing in-air handwriting (Xu et al., 2021). That work separated the recognition of the trajectory from the identification of the letters. In addition, Yanay & Shmueli (2020) followed a similar approach using smart-bands for identifying the trajectories through motion sensors. Other approaches used fingerprint detection for tracking air-writing in videos like in the work of Mukherjee et al. (2019). They concluded that the quality measured in frames per second was crucial for air-writing recognition. Although the current work is applied to handwriting in paper, some of the current findings could be applied later for one of the two parts of in-air handwriting recognition.

Another relevant sub-field is the handwriting analysis of patients of certain diseases. For instance, Diaz et al. (2021) proposed a mechanism for detecting Parkinson’s disease through the analysis of handwriting. In addition, Kamran et al. (2021) used deep learning for a similar purpose, aiming at achieving early detection of this disease for ameliorating the impact of this disease on patients avoiding unnecessary risky situations. This aligns with previous works about analyzing hand tremors while writing in smartphones (García-Magariño et al., 2016). It is worth noting that handwriting recognition could be more difficult in people with some diseases that can alter handwriting such as Parkinson with the symptoms of hand tremors. In this line of work, our approach could be relevant as it is robust to certain deformations through the use of coordinate vectors in 16 segments.

In the group of works for analyzing Arabic text, Haghighi & Omranpour (2021) presented a deep-learning model for recognizing digits in Arabic/Persian, using a conventional neural network in the first layer and a bidirectional long short term memory in the second layer. In this research line, Boufenar, Kerboua & Batouche (2018) presented an off-line deep learning method for recognizing Arabic characters with a deep convolutional neural network. They mentioned that deep learning was able to be applied only if enough data is available for each category and this was able to be achieved by using only isolated characters. Nahar (2018) applied combination of features for recognizing Arabic hand-writing. They used a combination of an artificial neural network with a genetic algorithm, and they also considered isolated characters. The current work aligns with this common trend but incorporates the novel use of coordinate vectors for recognizing Arabic hand-written Arabic text.

In conclusion, none of the aforementioned works uses coordinate vectors as an effective representation method for recognizing Arabic hand-written text. This gap of the literature is covered with the current work as presented in the next section.

## Method for handwriting recognition

**Figure 1:**
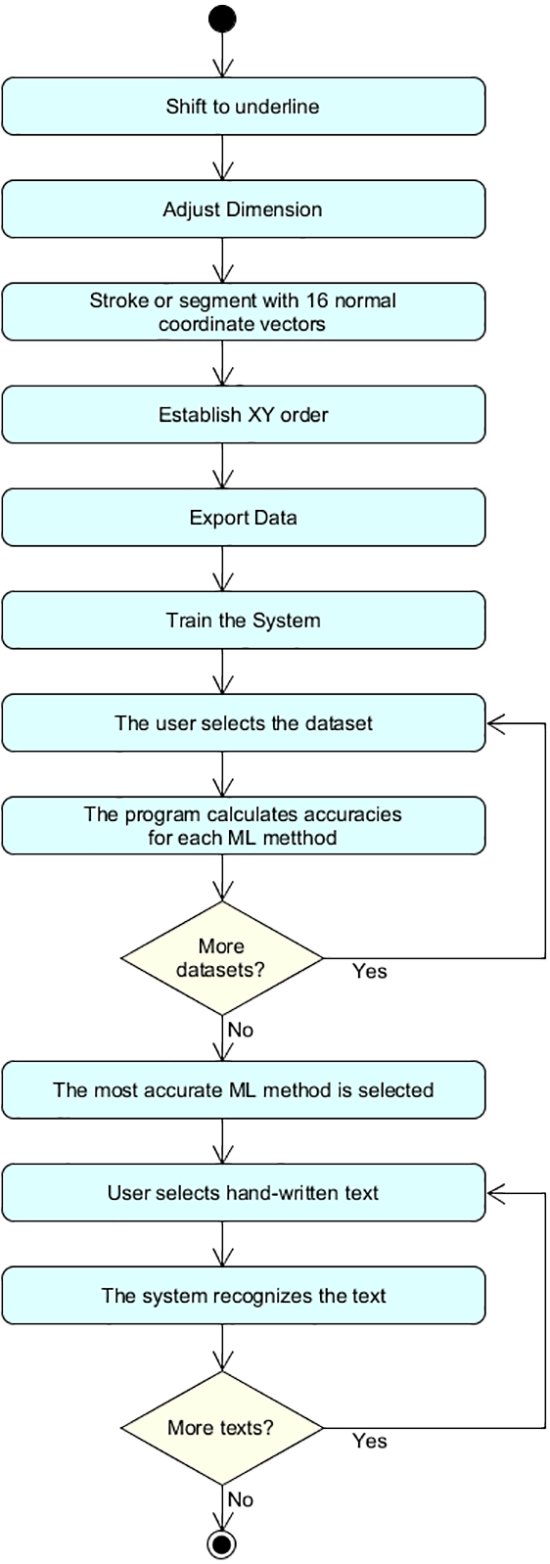
Block diagram.

**Figure 2:**
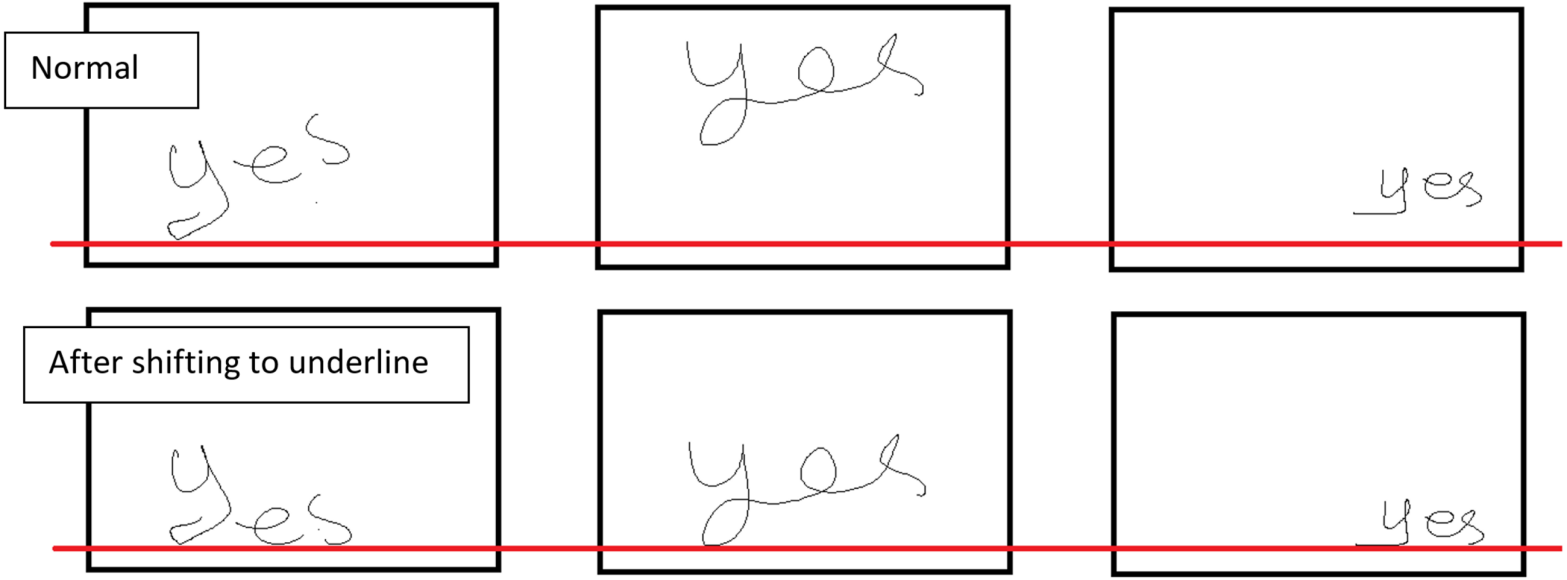
Shift to underline.

The proposed method for handwriting recognition involves the actions illustrated in the block diagram of Fig. 1. The first step is to shift the word to the corresponding underline. Figure 2 shows how the words are moved to the underline. In other words, we are moving the work a reference position. More concretely, in order to apply this shift, the algorithm selects the most bottom pixel of the word, and uses this as reference. This is useful for retrieving information from different sources and getting data fusion in Arabic handwriting recognition.

The second step is to adjust the dimension by scaling it to the reference size of 64x128 pixels, which is the common average size. The sizes of strokes vary, and in the proposed approach, the program interpolates all strokes to the size of 64 × 128 pixels, or Average Points Length (APL) for all datasets (file “interparc.m”). For example if we choose 64 pixels. the program will generate excel file with 128 columns, 64 columns for *x* coordinate and 64 for *y* coordinate.

**Figure 3:**
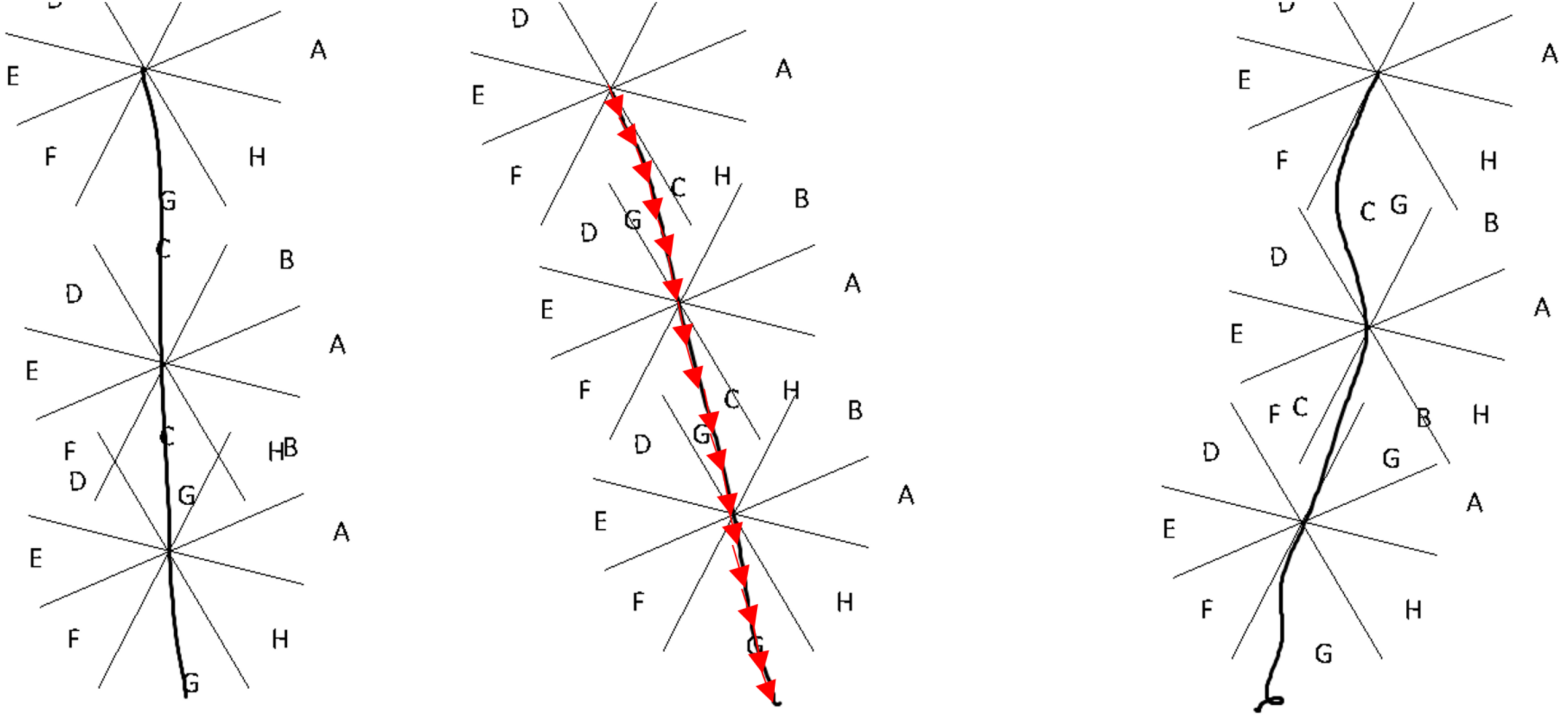
Visual explanation of rotation.

The next step is the application of the normal coordinate vectors considering 16 Segments. This step generates an excel file with normal coordinates. In regard to the 16 segments, in this case the dimension 64, 128 or APL will converted to 16-character string. Figure 3 illustrates three hand-written “l” letters. The first is vertical, the second with a slope to the right, the third with slope to the left but with defects. But if one wants to convert these three lines to 16 character string it will get “GGGGGGGGGGGGGGGG”. In this way, this approach can eliminate writer variations and styles, and many line defects in writing.

The idea is to distinguish the curvature of stroke using 16 slopes, but because that is impossible for human hand to write something every time as the first time, these 16 values will be vary from one attempt to another. Hence, the array of 16 slopes was replaced by array of 16 sectors. So the segment may take only eight directions not infinity number of values. This manner will dramatically reduce the different between two curvature strokes. The eight sector idea is an expansion to traditional chain code.

Because people tend to write using vertical and horizontal lines, the sectors were rotated 22.5 degrees. Experiments obtained better accuracy results after rotation than before rotation.

The next decision is to establish the XY order, in which the options are (a) “xy xy xy”’ or “‘xxx yyy”. In the former option, is one chooses 64 points from dimensions, the generated Excel file contains 128 columns: 64 x-coordinate and and 64 y-coordinate, their order x1-column, y1-column, x2-column, y2-column, …, x64-column, y64-column.

In the latter option, if 64 points are taken from dimensions, the generated excel file contains 128 columns: 64 x-coordinate and and 64 y-coordinate, and their order x1-column, x2-column, …, x64-column, y1-column, y2-column, ..., y64-column.

The final step depends on the recognition type, which can either be (a) writer identification or (b) pattern Recognition. If writer identification is chosen, an extra column is added to the generated excel file containing the writer name. If pattern recognition is chosen, the extra column in generated excel file contains the word itself using Latin letters.

### User interface

The user interface was designed for researchers with experience in ML with MATLAB for internal use of the research team. The user interface was developed following the principles of user-centered design with three expert researchers. They provided feedback in several iterations and their feedback was incorporated in the tool.

**Figure 4:**
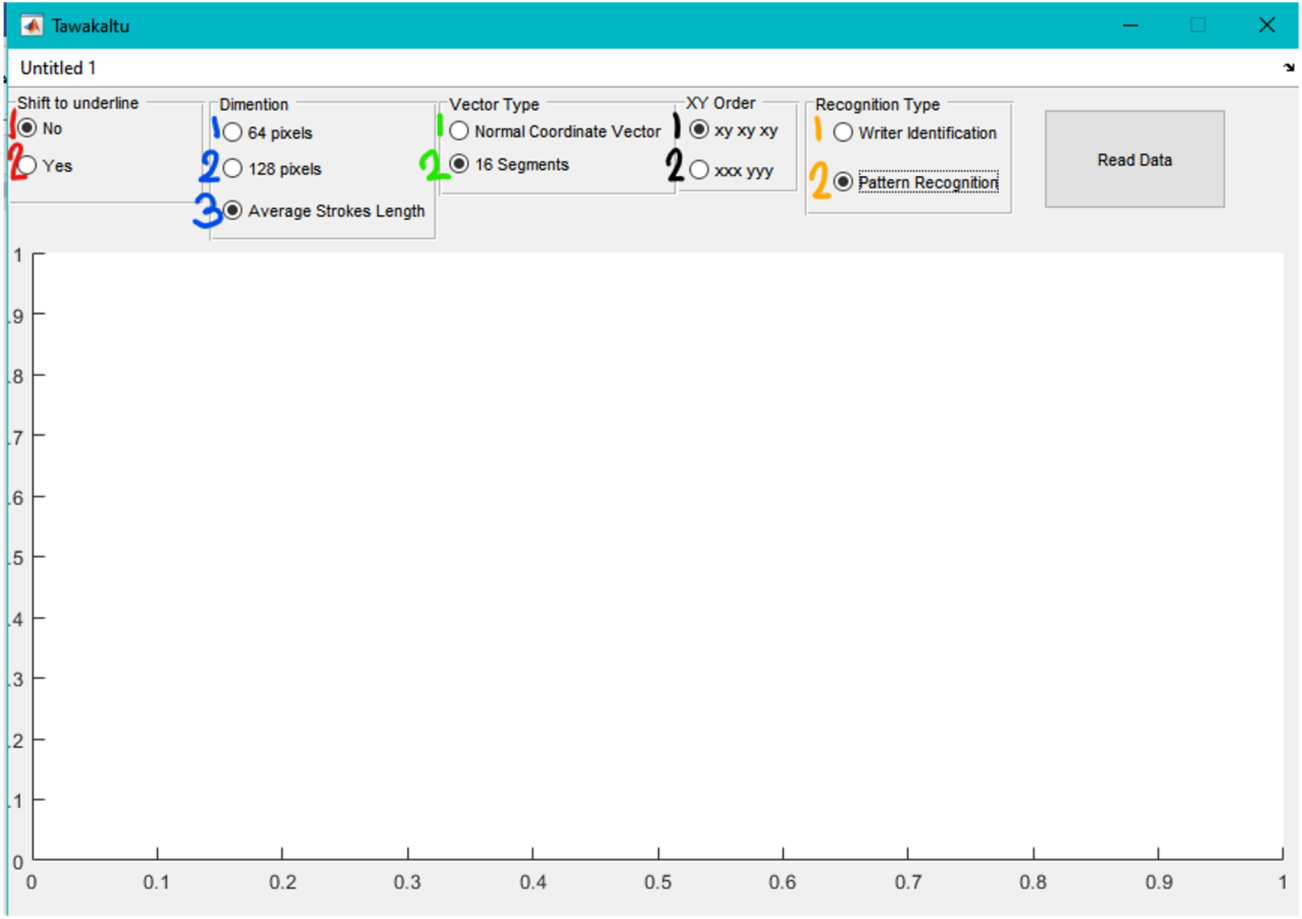
First step in user interface.

**Figure 5:**
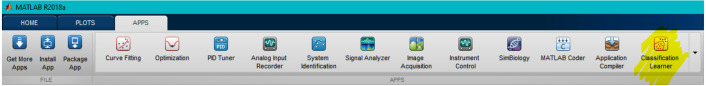
Second step in user interface.

**Figure 6:**
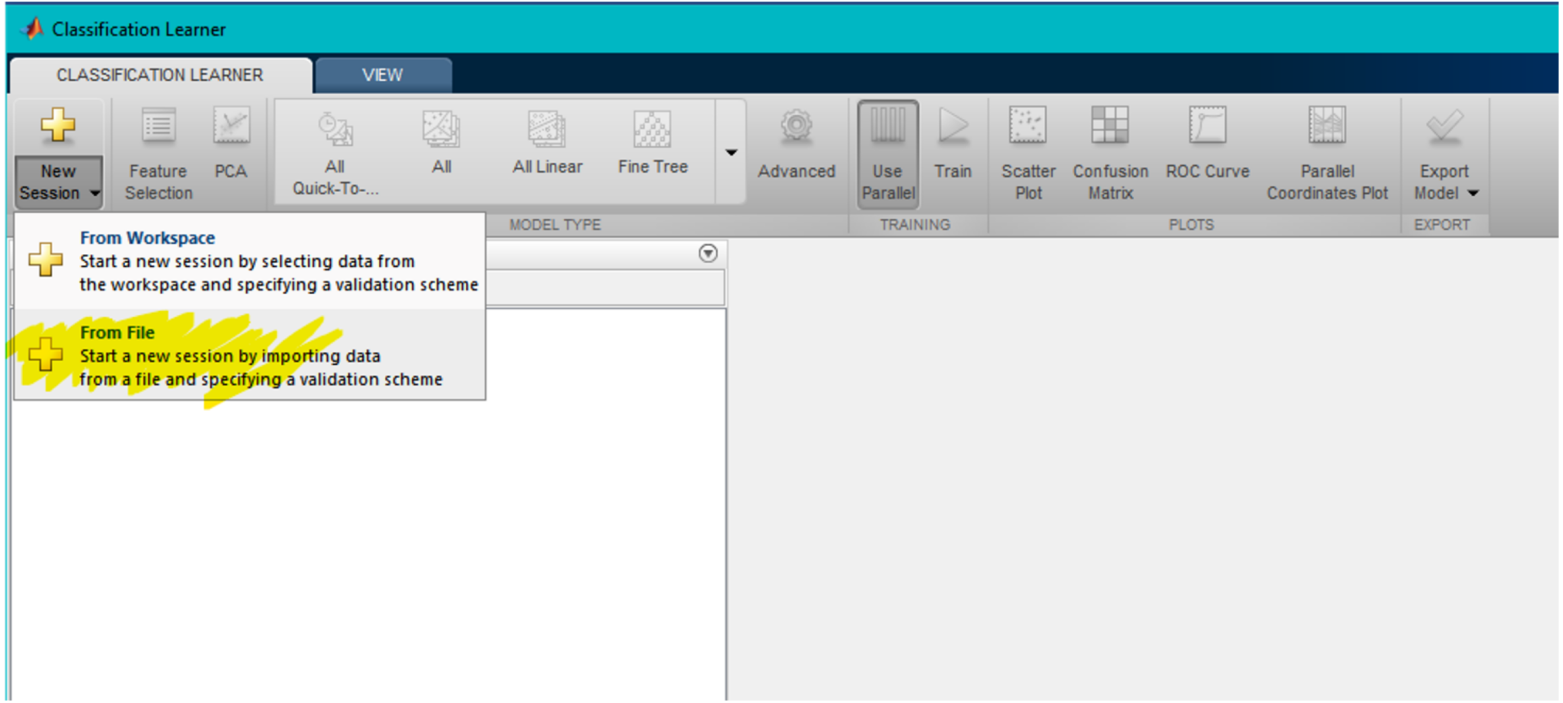
Third step step in user interface.

This approach can be executed with MATLAB with the source code developed in this work. The steps for the execution are the following ones:

1. Run “tawakaltu.m” file and one will see Fig. 4.
2. Choose the combination you want (from 2 × 3 × 2 × 2 × 2 = 48 possible combinations)
3. Press Read Data then choose the dataset (62 × 1 or 6 × 10).
4. After some time (10–20 min in common laptops) excel file will be generated. The name of this file denote the choose combination, like Data__1(No)_3(ASL)_2(16S)_1(XYXY) _2(PR)__.xlsx.
5. From MATLAB, run classification learner toolbox, as one can observe in Fig. 5.
6. From classification learner, the user should click on “New Session”, and then click on “From File” in the dropdown list, as shown in Fig. 6.

## Results

The experimentation was conducted following the steps mentioned in ’User Interface’. As result, we could have obtained 48 files for each dataset, but because of time consuming, we excluded “XY order” group, so we got only 24 files for each dataset (that’s it: “xy xy xy” only used, “xxx yyy” not used).

Researchers can reproduce the same test obtaining the same excel file, but result of classification learner can be slightly different because of the stochastic component of some ML approaches.

**Figure 7:**
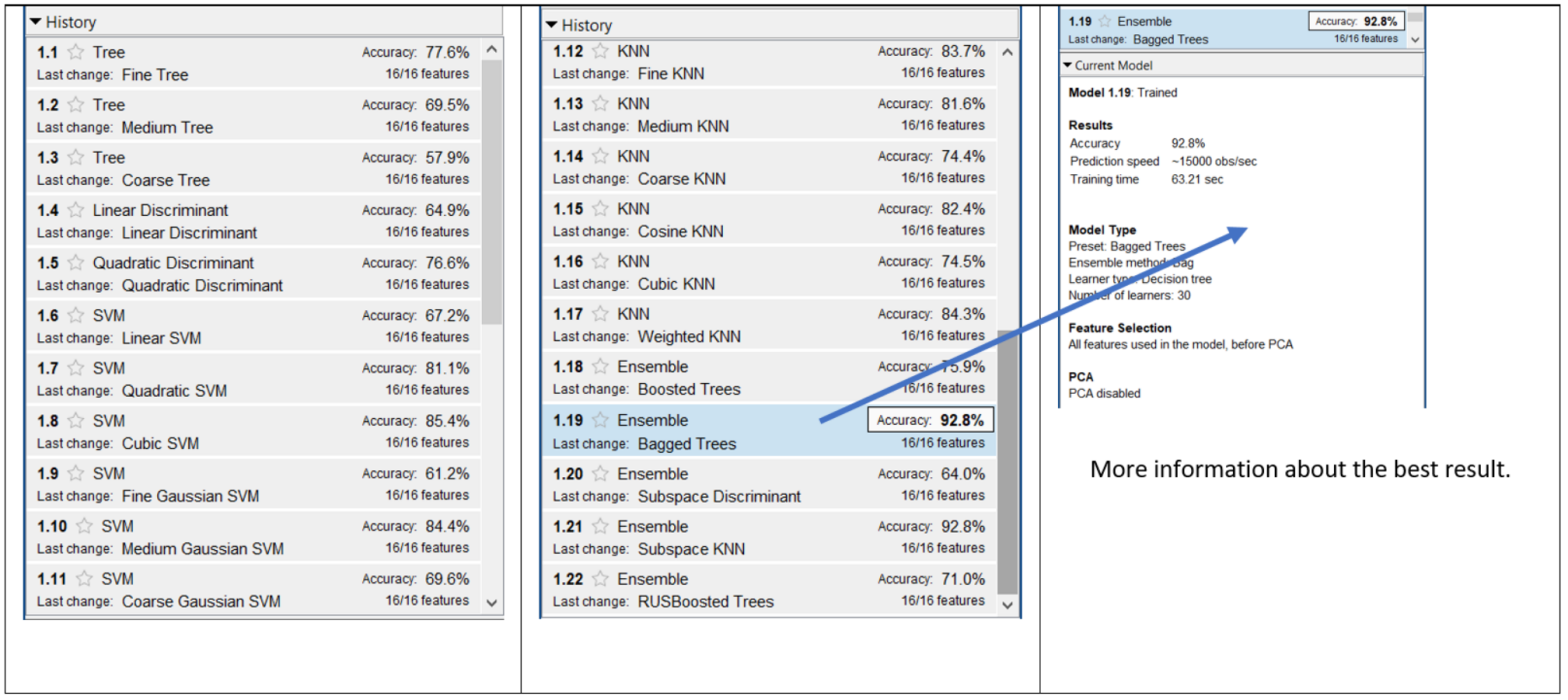
Result of 13,212 combinations for 6 × 10 dataset.

Figure 7 shows the results of the 13,212 combinations for the 6 × 10 dataset. One can observe tat the highest accuracy was obtained with (a) the Ensemble and Bagged Trees and (b) Ensemble with Subspace K-nearest Neighbors (KNN). Both cases obtained an accuracy of 92.8%. The lowest accuracy was obtained with the combination of Tree and Coarse Tree with a value of 57.9%. The average of accuracies of this dataset was 76.0% and the standard deviation (SD) was 9.43%.

**Figure 8:**
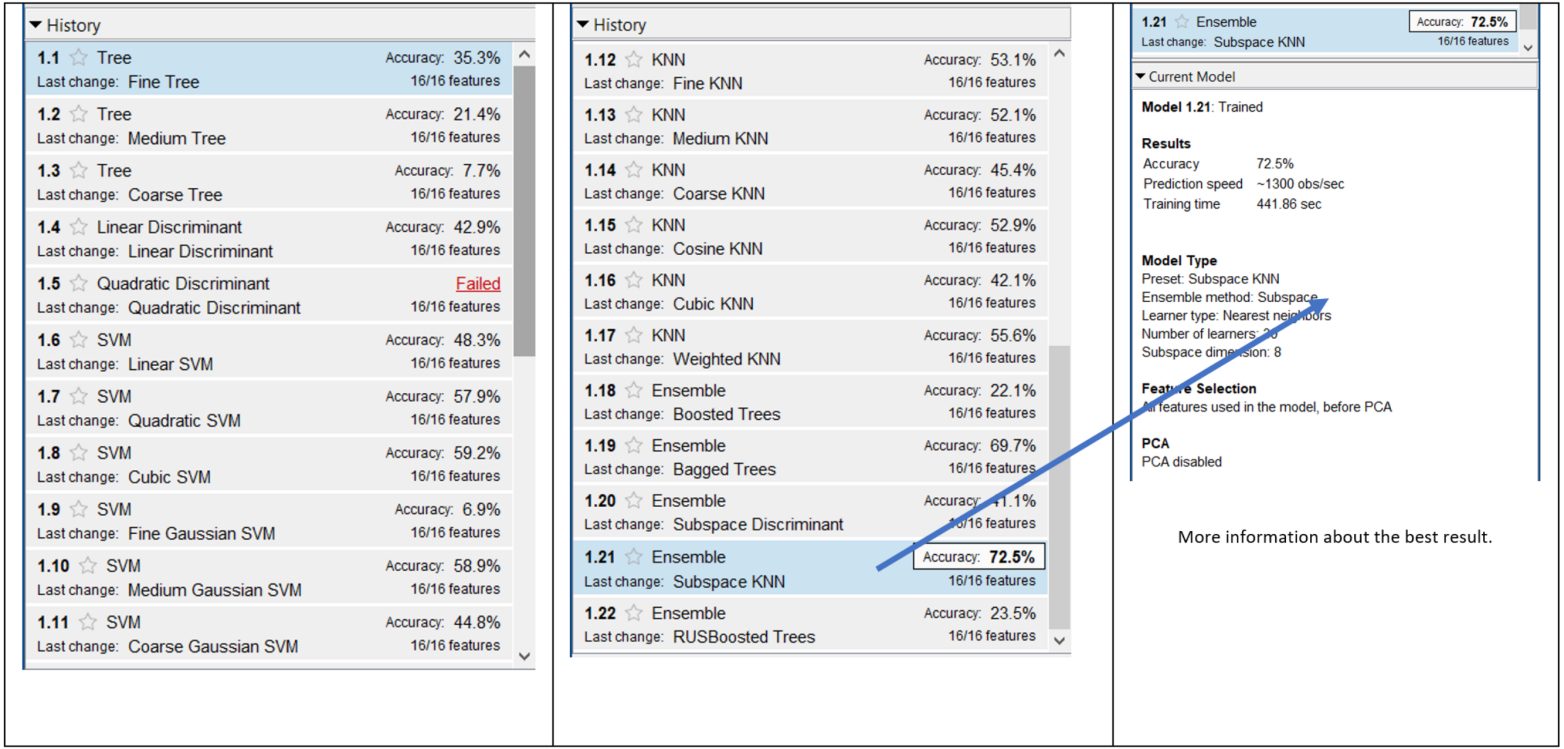
Result of 13,212 combinations for 62 × 1 dataset.

Figure 8 presents the results of 13,212 combinations for 62 × 1 dataset. The accuracy results were generally lower and the best accuracy was obtained by Ensemble and Subspace KNN with a value of 72.5%. The lowest accuracy was obtained with Tree and Coarse Tree. The average of accuracies was 43.5% for this dataset with a SD of 17.86%.

**Figure 9:**
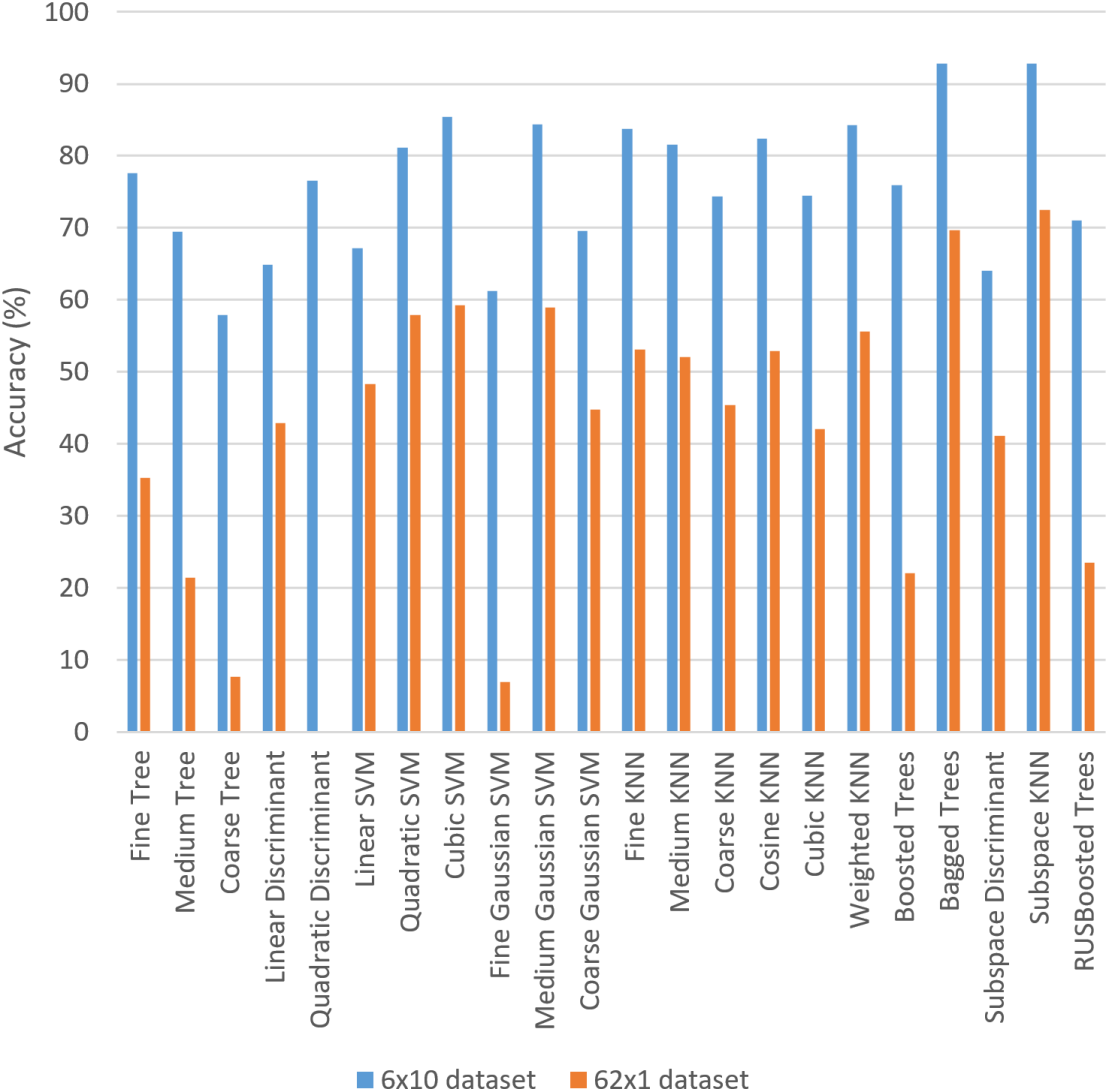
Comparison of the results for the ML methods and both datasets.

In order to facilitate the comparison, Fig. 9 shows a graphical comparison among all the ML methods for both datasets. It is worth highlighting that the worst combination (*i.e.,* Tree and Coarse Tree) is the same in both datasets. One of the best accuracies is obtained with the same combination (*i.e.,* Ensemble and Subspace KNN) is the same for both datasets.

## Discussion

In the light of the results of this work, we observe that the selection of an appropriate dataset is key for obtaining a high accuracy. This explains the accuracy differences between the different datasets. This aligns with previous findings in the field of hand-writing recognition (Varalakshmi, Negi & Krishna, 2012). In fact, they not only observed this change accuracy for different dataset, but also explained guidelines for obtaining right datasets for text recognition. They even proposed a way of automatically generating datasets. In this line of work, we plan to design new datasets and evaluate them in order to assess if the accuracy could be improved further by using other datasets. In this way, we have tested data fusion and we will further evaluate data fusion in this planned enhanced experimentation.

If we further compare the results in both datasets, we observe that the accuracy was lower in 62 × 1 dataset than in 6 × 10 dataset for all the ML combinations. Thus, apparently 6 × 10 is much more relevant for training all the 22 combinations. However, the SD of accuracies is higher in 62 × 1 dataset than in the other dataset and consequently the variation of results is considered to be higher. Thus, this corroborates that 6 × 10 is better for training the system in this context. The 6 × 10 dataset has probably obtained much higher accuracies than 62 × 1 dataset since the former one uses more information for training each class, and the number of classes is lower. The amount of information is usually known to be directly relevant in the ML classifiers (Heilman, Kaefer & Ramenofsky, 2003). In addition, the classifiers with higher number of classes usually obtain lower accuracy values than classifiers with lower number of classes, as commonly stated when comparing classifiers in different domains (Read et al., 2021). The bagging methodologies were also found especially relevant in the improved recognition results of medieval hand-written Gurmukhi manuscripts (Kumar et al., 2019), aligning with the highest accuracy results obtained in our approach with the bagged trees.

Observing further the details of the specific results of the different ML methods (presented in Fig. 9), all the seven KNN methods obtained more than 40% of accuracy even in the 62 × 1 dataset, and this property is not so common in all the other combination. Thus, we hypothesize that finding the most similar cases and providing the corresponding solution guarantees a minimum of accuracy in Arabic handwriting, aligning with the findings in other works that use KNN (Shokrzade et al., 2021). Furthermore, we hypothesize that the reason that bagged trees obtained the highest accuracy is that certain features in Arabic handwriting letters (Al-Hadhrami et al., 2015) are adequate for classifying the samples following the common decision structure in decision trees.

Coordinate vectors have been useful for identifying different hand-written styles with different inclinations, as shown in the current experimentation. This aligns with previous finding for the Malayalam language (Joseph & Hameed, 2014), although in this work they mainly tested their approach with straight handwriting rather using texts with different inclinations. Thus, the proposed approach has shown the utility of coordinate vectors for handwriting recognition in texts with different inclinations.

The different ML methods obtain similar results in most cases. For example the extremes of the highest and lowest accuracies are the same in both datasets. They also keep coherence in most cases, showing that the findings about ML methods applied in handwriting recognition are consistent through different datasets and may be relevant for other researchers. It is worth highlighting that consistency through different datasets has been considered important in the ML literature (Moore & Lee, 1998) to provide support for relevant research. In fact, in ML review papers usually gather results from different researchers in different datasets to identify relevant aspects in specific fields.

The authors originally selected MATLAB as the original target users were familiar with the environment associated with this language. However, this language has the drawback that the necessary Integrated Development Environment (IDE) is not free, and requires that users had bought the corresponding license. For this reason, we are planning to migrate the system to Python programming language using Scikit-learn framework, which provides the necessary support for the ML methods required in our approach. In this way, the proposed approach will be freely available for more potential users.

In order to facilitate the usage of this approach, we plan to develop an easy-to-use online web interface for using the proposed system for analyzing images with Arabic hand-written text and accessing to the information from the previously analyzed hand-written text. This will be implemented with the Bottle framework over the aforementioned Python implementation, using the Paste server for ensuring stability of the web application server. This will openly provide the service to anyone without needing any specific knowledge of programming or pattern recognition. This system will be also useful for collecting feedback from a wide range of users, which can help in improving the system.

Although the techniques of OCR now obtain high accuracy results (Mei et al., 2018) reducing error rates, it is well known that these techniques obtain much lower accuracy rates when applying them on handwritten text (Pramanik & Bag, 2018). Furthermore, Arabic handwriting is especially difficult to automatically decompose in characters given its particularities (Jayech, Mahjoub & Amara, 2016). We discarded using the common OCR techniques in our approach for all these reasons.

## Conclusion and future work

This work has presented a novel approach for analyzing hand-written text with coordinate vectors using 16 different segments. The implementation of this approach in MATLAB has shown its potentiality in analyzing datasets with different writing inclinations. This has been useful for applying data fusion considering different sources with different handwriting styles.

We plan to enhance the evaluation of this approach by performing quantitative analysis with other existing approaches to further the improvement over the existing alternatives. This evaluation will use a common dataset for both training and validation, considering a greater range of writing styles with different inclinations. Furthermore, we plan to collect data from new users in Arabic text recognition by providing a web interface for collecting data. This online system will provide a service that will motivate the usage of the system, and will implicitly gather data following the European policies regarding data protection. This will help to identify opportunities for improving the system and the corresponding algorithm.

##  Supplemental Information

10.7717/peerj-cs.705/supp-1 Supplemental Information 1 All programming code in Matlab

## Additional Information and Declarations

### Data Availability

The following information was supplied regarding data availability: All the MATLAB programming code is available in the Supplemental File.
